# Factors associated to patients’ referral to public or private Covid-19 healthcare centers in Tabriz, Iran

**DOI:** 10.1186/s12913-023-09640-z

**Published:** 2023-06-13

**Authors:** Nasrin Jafari, Hossein Akbari, Parvin Sarbakhsh, Abbasali Dorosti, Simin Khayatzadeh, Asghar Mohammadpoorasl

**Affiliations:** 1 Department of Statistics and Epidemiology, Faculty of Health, Tabriz University of Medical Sciences, Tabriz, Iran; 2 Department of Anesthesiology, Tabriz University of Medical Sciences, Tabriz, Iran; 3 East Azerbaijan Province Health Center, Tabriz University of Medical Sciences, Tabriz, Iran

**Keywords:** Public Sector, Private sector, Contact tracing, Patient preferences, SARS-CoV-2

## Abstract

**Background:**

In Iran, tracking of patients and its associated data recording in private healthcare centers are poor, and thus a majority of patients suffering from Covid-19 are treated without any control on the isolation and quarantine processes. The present study aims to investigate the factors contributed to referral to private or public healthcare centers that provide Covid-19 care services.

**Methods:**

This cross-sectional study was conducted from November 2021 to January 2022 in Tabriz, Iran. We invited a total of 258 and 202Covid-19 patients from governmental and private healthcare centers, respectively, to participate in the study by convenient sampling method. Applying a self-administered questionnaire, we collected data on the reason of referring to the healthcare centers, patient’s waiting time, quality of healthcare services received by the patients, patients’ level of satisfaction, accessibility, insurance coverage, perceived severity of the disease, and the level of staff compliance from health protocols. Logistic regression model was used for data analysis by using SPSS-26 software.

**Results:**

Adjusted for other variables, higher socio-economic status (AOR (Adjusted Odds Ratio) = 6.64), older age (AOR = 1.02), referral of friends and family members (AOR = 1.52), shorter waiting time (AOR = 1.02) and higher satisfaction (AOR = 1.02) were contributed to referral to private centers. Better accessibility (AOR = 0.98) and wider insurance coverage (AOR = 0.99) were also contributed to referral to governmental centers.

**Conclusion:**

Providing more appropriate insurance coverage by private healthcare centers, and promoting their level of accessibility seems to promote patients’ referral to such centers. Moreover, establishing an accurate system for recording patients’ information and follow up in private centers might promote the role of private healthcare centers in managing the overload of patients on healthcare system during such epidemics.

## Background

Covid-19 has resulted in various problems in the health systems of the countries, worldwide. The demand for Covid- 19 diagnostic and treatment services has also increased significantly from both public and private healthcare providers [1]. The major principles of managing the Covid-19 pandemic are as follows: [1] identifying and following up the patients and individuals who come in contact with them, and [2] recording and reporting their associated statistics, accurately and electronically. In health system, identifying, contact tracing, also recording the statistics of subjects should be completed to better management and disease control.

It could be noted that, in public health care centers, the information of some of Covid-19 cases with either probable or suspect symptoms are recorded in electronic health records during active case finding and/or screening process. This process allows to record statistics, and to track the patients and the people who they were in contact with [2, 3]. In the Iranian private healthcare centers the reporting process of contagious diseases are less efficient [4]. Therefore, in private sector, majority of probable and/or suspect Covid 19 cases are lost to follow-up. So, patients suffering from Covid-19 are treated without any control on the isolation and quarantine process of those around them.

According to the studies, in general, private sector costs are higher than the public sector and more than 95% of payments to the private sector are out-of-pocket payments [5]. But what are the associated factors for choosing the private sector by Covid-19cases?Several previous studies have investigated the determinants of referring to private and/or public healthcare centers. In a study conducted by Lankarani et al., distance from healthcare centers, time to admission, waiting time in the clinic, and security were identified as effective factors on patients’ satisfaction with private healthcare centers. One of the factors that caused dissatisfaction with the services provided by the private centers was high cost [6]. In another studies, age, economic status of the family, high accessibility to highly-experienced and reliable specialists, and cleanliness of hospital environment were reported as the determinants of choosing and referring to either a private or public health care center [7–9]. To the best of our knowledge, no study has yet been conducted on the determinants of Covid-19 patients’ referral to private and/or public healthcare centers in a developing country, like Iran. So, our aim in the present study was to investigate the factors contributed to patients’ referral to private and/or public healthcare centers that provide Covid-19 care services in Tabriz, Iran.

## Methods

This cross-sectional study was conducted from November 2021 to January 2022 in Tabriz, Northwest of Iran. One governmental hospital and two governmental healthcare centers (as governmental sector), and one private hospital, two clinics and one university-affiliated clinic (as private sector) were selected as our data collection centers. Applying convenient sampling, 460 suspected, probable and/or confirmed cases of Covid-19 patients referring to the aforementioned centers, participated in the study.

The sample size was determined according to the main purpose of the study, which is to determine the factors influencing the selection of service providers. In order to have reliable estimates in logistic regression, the number of subjects per variable should be at least 10. Considering that about 20 variables were considered in the present study, according to the rule of thumb, 200 samples from each group were needed. The number of study sample in this research was 202 subjects in the private sector and 258 subjects in the public sector.

### Instrumentation

Applying a researcher-made questionnaire, the view points of the patients on the following questions were asked:

*The reason of referring to the healthcare center* was assessed using the following item: Why did you refer to this center? The following choices were presented as the possible responses: (A) I have the symptoms of Covid-19, and referred here to receive treatment; (B) I have been around a person with/suspected of Covid-19, and I referred to this center for testing; (C) I need to receive a doctor’s certificate, and a Covid-19 test result to be presented to my place of work.

*The history of referral to the healthcare center* was assessed using the following question: Have you and your family members and/or friends/acquaintances come to this center to receive Covid-19 services, previously?

*Level of staff compliance from health protocols by 4 questions*, *quality of healthcare services received by the patients by 7 questions*, *accessibility by 7 questions*, and each items of patients’ *satisfaction*, *waiting time*, *insurance coverage*, and *perceived severity of the disease* were measured by one-item scales. Response format for all these questions was based on a Likert-type scaling: very high [5], high [4], medium [3], low [2] and very low [1]. Higher score in each item demonstrated more positive viewpoint on choosing the centers for receiving Covid-19 treatment services.

Normalization is a technique often applied as part of data preparation. The goal of normalization is to change the values of numeric columns in the dataset to a common scale, without distorting differences in the ranges of values. In our study, because the number of questions in each subscale was different, in order to equalize the range of scores and make them comparable, the scores of each subscale were normalized and the resulting number was multiplied by 100.

In order to develop the questionnaire, we firstly listed a set of factors affecting people’s referring to health centers, after a review on literature. In the next step, several individual interviews were conducted with two groups of people, including Covid-19 patients referring to health centers, and a group of physicians and specialists working in Covid-19 services wards. Their ideas and comments were used to improve the necessity and clarity of the items. This initial draft of the questionnaire was sent to a group of experts so as to assess its content validity. Obtaining and evaluating of experts opinions were qualitative. After applying the opinions of the experts, we evaluated the final scale in terms of reliability and face validity in a pilot sample of people (n = 18). Cronbach’s alpha was used to assess internal consistency of the scale (α = 0.881).

Data on demographic characteristics of study participants, including age, gender, marital status, level of education and socio-economic status (SES), were also collected by using a questionnaire. Principal component analysis (PCA) was used to measure SES of the participants. Participants’ education, household assets and income were included in the PCA to measure SES. Accordingly, the participants were divided into low, middle and high SES groups.

Data collection was performed by self-completion of the questionnaire in majority of participants. In some cases with low literacy level, face-to-face interview by trained and qualified interviewer was done to complete the questionnaire.

### Data analysis

Independent sample t-test and Chi-square tests were used to investigate the mean of quantitative, and the nominal/scale variables, respectively. Logistic regression is used to investigate the effect of predictor variables on binary dependent variable. In our study, the response variable was the choice of a private or government center by patients and the determination of factors related to this choice, so logistic regression was used to investigate this relationship. Variables with a P-value of < 0.2 in the bi-variable model, were entered into the multivariable model. Level of significance was considered to be 0.05, a priori. Data analysis was performed using SPSS v. 26 software.

## Results

In total, 258 (56.1%) respondents from governmental centers and 202 (43.9%) respondents from private centers were included in the study. The mean age of participants was 40.1 ± 14.5 years old (Min: 10, Max: 80). More than half of the participants were male (55.9%) and 69.0% of them was married.

Demographic characteristics of the participants, the reasons for their referral, and the mean score of the scales are represented in Table 1.Significant differences were found in the patients referring to governmental and private centers by age, socio-economic status, patients’ waiting time, insurance coverage, and disease severity.

**Table 1:** Differences in respondents’ demographic characteristics and scales’ items by type of centers

	Private sector N = 202	Governmental sector N = 258	P-value
**Age (Mean (SD))**	42.9 (15.2)	38.8 (14.1)	0.004
**Gender (n (%))**			
Male	108 (54.0)	149 (57.8)	0.422
Female	92 (46.0)	109 (42.2)	
**Marital status (n (%))**			
single	54 (28.0)	63 (24.7)	0.287
married	126 (65.3)	183 (71.8)	
Other	13 (6.7)	9 (3.5)	
**Socioeconomic status (n (%))**			
Low	33 (17.8)	112 (48.9)	< 0.001
Middle	65 (35.2)	79 (34.5)	
High	87 (47.0)	38 (16.6)	
**Reasons to visit the centers (n (%))**			
*Having covid-19 symptoms and receiving treatment*	*101 (59.1)*	*122 (53.3)*	0.388
Having exposure to Covid-19 cases, and need to be tested	49 (28.7)	69 (30.1)	
Need a doctor’s certificate	21 (12.2)	38 (16.6)	
**Referral of friends and family to a private center**			
No	69 (39.7)	105 (60.3)	0.013
Yes	126 (52.5)	114 (47.5)	
**Patients’ Waiting time(Mean (SD))**	84.4 (24.6)	73.0 (27.7)	< 0.001
Level of staff compliance from health protocols**(Mean (SD))**	76.6 (18.5)	75.7 (21.8)	0.628
Quality of healthcare services received by the patients**(Mean (SD))**	76.1 (19.8)	75.5 (20.2)	0.748
**Level of satisfaction (Mean (SD))**	65.7 (36.1)	66.2 (33.9)	0.889
**Accessibility (Mean (SD))**	60.2 (18.9)	63.6 (20.5)	0.071
**Insurance coverage(Mean (SD))**	59.6 (35.9)	75.3 (26.3)	< 0.001
Perceived severity of the disease**(Mean (SD))**	42.6 (35.9)	60.6 (30.3)	< 0.001

According to the bi-variable model, and after adjustment for confounding variables, age and socio-economic status of the patients were significantly associated to referring to private centers. In both the bi-variable model and multivariable model, the probability of referral of friends and family members to private centers increased significantly, but this relationship was statistically significant only in the bi-variable model. Waiting time was significantly contributed to the probability of referring to private centers, in both bi-variable model (OR: 1.02) and multivariable model (AOR: 1.02) models. Accessibility (AOR: 0.98) and insurance coverage (AOR: 0.99) were also associated to referring to governmental centers. Level of satisfaction (AOR: 1.02) and perceived severity of the disease (AOR: 0.99) increased the probability of referring to private and governmental centers, respectively (Table 2).

**Table 2:** Logistic regression model for referring to a private center

	Bi-variable model		Multivariable model	
	OR (95% CI)	p-value	AOR (95% CI)	p-value
Age	1.02 (1.01 to 1.03)	0.005	1.02 (1.00 to 1.04)	0.014
Socio-economic status				
Low	Ref.			
Middle	2.80 (1.68 to 4.64)	0.006	2.38 (1.36 to 4.17)	0.002
High	7.77 (4.51 to 13.39)	< 0.001	6.64 (3.68 to 11.99)	< 0.001
Referral of friends and family to a private center				
No	Ref.			
Yes	1.68 (1.13 to 2.50)	0.010	1.52 (0.92 to 2.57)	0.105
Patients’ Waiting time	1.02 (1.01 to 1.03)	< 0.001	1.02 (1.01 to 1.03)	0.002
Level of staff compliance from health protocols	1.00 (0.99 to 1.01)	0.635	-	-
Quality of healthcare services received by the patients	1.00 (0.99 to 1.01)	0.747	-	-
Level of satisfaction	1.00 (0.99 to 1.01)	0.889	-	-
Accessibility	0.99 (0.98 to 1.00)	0.075	0.98 (0.97 to 1.00)	0.019
Insurance coverage	0.98 (0.98 to 0.99)	0.001	0.99 (0.98 to 1.00)	0.007
Perceived severity of the disease	0.98 (0.98 to 0.99)	< 0.001	0.99 (0.98 to 1.00)	0.012

## Discussion

The aim of this study was to investigate the factors contributed to the referral of Covid-19 patients to private or public healthcare centers in Tabriz, Iran. High socio-economic status, older age, referring to the center by friends and/or family members, shorter waiting time and higher satisfaction were contributed to patients’ referral to private centers. Better accessibility, wider insurance coverage, and perceived severity of the disease were associated to patients’ referral to governmental centers.

According to our findings, the Covid-19 patients with higher socio-economic status often referred to private healthcare centers. This finding was similar to those reported by Zhu et al., who indicated that people with higher income mainly choose to refer to private healthcare centers [10]. Besides, several previous studies have also reported that patients with higher socio-economic status choose referring to private sector services, and people with lower socio-economic status choose referring to public sector services [11, 12].

In the present study, older age increased the possibility of referring to private centers, while in previous studies, the majority of younger patients preferred to refer to private healthcare centers [10, 12]. In the study conducted by Zhu et al., younger patients often preferred to refer to private centers with high quality and low waiting time [10]. In the study performed by Gil et al., older age increased the chance of referral to governmental healthcare centers [12]. It seems that in the Iranian society, as age increases, the socio-economic status of people improves, and hence, they could pay higher medical costs of private centers, in exchange for receiving high quality services.

In our study, low waiting time and higher levels of patients’ satisfaction also increased the possibility of referring to private healthcare centers among patients with Covid-19.Similarly, Verma et al., found waiting time and satisfaction with service provider to be determining factors for referring to such centers [13]. It seems that a majority of patients choose to refer to private healthcare centers because of reduced waiting time in such centers [14]. Another study also declared long waiting time as one of the concerning issues when referring to governmental healthcare centers [15].

High level of accessibility, high availability of diagnostic laboratory equipment, wide insurance coverage, and perceived severity of the disease were identified as perceived barriers of our respondents’ referring to private healthcare centers. According to a previous study, short distance and better insurance coverage were among the determining factors of choosing a health center [11]. In another study conducted by Oredola et al., more than half of the patients referred to governmental hospitals, due to their proximity and closeness. Also, patients preferred public centers when their diseases were severe [14]. In the study performed by Nonvignon et al., patients with insurance coverage used public healthcare services more than private ones [16].

Present study is one of the few studies that investigated the factors contributed toCovid-19 patients’ referral to private healthcare centers during the pandemic. Limitations of this study were as follow: [1] the data were collected applying a self-report questionnaire, which warrants recall bias [2] Given the cross-sectional nature of the study, the outcomes of the present study only indicate the relationships between the variables, and thus causal inferences are warranted.

## Conclusion

Older age, higher socio-economic status, lower levels of waiting times for service provision, and higher levels of patient satisfaction were identified as determinant factors for patients’ referral to private healthcare centers. On the other hand, higher levels of accessibility to governmental healthcare centers and wider health insurance coverage reduced the possibility of referring to the private centers.

Providing more appropriate insurance coverage by private healthcare centers, and promoting their level of accessibility seems to promote patients’ referral to such centers. Moreover, establishing an accurate system for recording patients’ information and follow up in private centers might promote the role of private healthcare centers in managing the overload of patients on healthcare system during such epidemics.

## Data Availability

The datasets used and/or analyzed during the current study are available from the corresponding author on reasonable request.

## List of abbreviations

PCA: Principal component analysis
SES: Socio-economic status
